# Cervicovaginal zinc and copper profiles across vaginitis phenotypes and hydrogel assignment: an exploratory pilot study

**DOI:** 10.1186/s12905-026-04768-6

**Published:** 2026-08-01

**Authors:** Alexandra Kunkli, Dávid Rátonyi, Barbara Kozma, Bálint Farkas, László Majoros, Robert Póka, Attila G. Sipos, Peter Takacs, Zoárd T. Krasznai, Bence Kozma

**Affiliations:** 1 Faculty of Medicine, Department of Obstetrics and Gynecology, University of Debrecen, Nagyerdei krt. 98, Debrecen, 4032 Hungary; 2 Doctoral School of Nutrition and Food Sciences, University of Debrecen, Egyetem tér 1, Debrecen, 4032 Hungary; 3 Faculty of Medicine, Department of Medical Microbiology, University of Debrecen, Debrecen, Hungary; 4 Department of Obstetrics and Gynecology, Division of Female Pelvic Medicine and Reconstructive Surgery, Eastern Virginia Medical School, 825 Fairfax Avenue, Suite 526, Norfolk, Virginia 23507-2007 USA

**Keywords:** Zinc-containing hydrogel, Functional hydrogel, Vulvovaginitis, Cervicovaginal lavage, Zinc, Copper

## Abstract

Zinc (Zn) and copper (Cu) support epithelial integrity, nutritional immunity, and host–microbiome interactions, yet cervicovaginal trace-metal dynamics across vaginitis phenotypes remain poorly defined. We prospectively quantified cervicovaginal lavage (CVL) Zn and Cu by ICP-OES in an exploratory secondary analysis nested within a single-center randomized trial involving 56 symptomatic women and asymptomatic controls (mean age, 36.0 ± 11.8 years; mean BMI, 23.1 ± 4.9 kg/m²). We examined whether CVL trace-element concentrations varied according to culture-defined phenotype and randomized hydrogel exposure. CVL Zn concentrations were higher in the Candida albicans-positive (120.2 ± 95.6 µg/L) and symptomatic culture-positive bacterial phenotypes (83.8 ± 55.2 µg/L) than in controls (58.6 ± 27.2 µg/L), whereas Cu showed less consistent variation. Among participants assigned to hydrogel, phenotype-specific differences from controls were not statistically significant. In the fluconazole-only group, higher Zn concentrations were observed in the C. albicans-positive phenotype (154.3 ± 113.5 vs. 54.2 ± 23.8 µg/L), accompanied by higher Cu concentrations (12.13 ± 4.76 vs. 5.76 ± 2.86 µg/L). These exploratory findings indicate that cervicovaginal trace-element profiles may vary across symptomatic culture-defined phenotypes and treatment groups. Our findings should be interpreted as hypothesis-generating. Biological and clinical relevance remains uncertain and warrants confirmation in adequately powered, longitudinal studies using dilution-normalized CVL measurements. Trial registration: The study protocol was approved by the Hungarian National Institutional Review Medical Research Council (approval No. 3282-7/2023/EUIG). The parent trial is registered at ClinicalTrials.gov (NCT05895162; 2023-05-15). Each participant signed a written informed consent form. All procedures performed in studies involving human participants were under the ethical standards of the Hungarian National Institutional Review Medical Research Council and with the 1964 Helsinki Declaration and its later amendments or comparable ethical standards. This article does not contain any studies with animals performed by any of the authors.

**Supplementary Information:**

The online version contains supplementary material available at https://doi.org/10.1186/s12905-026-04768-6.

## Introduction

Vaginitis is a clinical syndrome characterized by discharge, pruritus, irritation, malodor, and dyspareunia that reflect disruption of epithelial barrier function, the vaginal microbiota, and mucosal immunity. The major etiologies are bacterial vaginosis, vulvovaginal candidiasis, and Trichomonas vaginalis infection, although noninfectious causes also occur, with presentation influenced by sexual activity, hormones, antibiotic exposure, and host factors [1, 2]. Vulvovaginal candidiasis (VVC) is a leading cause of symptoms in practice. While most episodes respond to azoles, recurrent VVC frequently requires prolonged suppressive therapy (e.g., weekly oral fluconazole for months) and relapse after discontinuation is common. These shortcomings underscore the need for adjunct, mucosa-targeted strategies that stabilize the local environment and support longer-term symptom control alongside antifungal therapy [3–5].

Metal homeostasis is governed by tightly regulated networks controlling uptake, utilization, storage, and export. In vertebrates, tissue metal levels largely reflect intestinal absorption, whereas bacterial pathogens must acquire essential metals from host niches during infection [6]. Zinc (Zn), the second most abundant trace element after iron, is indispensable for growth and immune competence [7]. As a cofactor for numerous enzymes and transcription factors, Zn supports DNA synthesis, antioxidant defense, and immune signaling [8], and deficiency is associated with impaired host defense and delayed tissue repair [9]. Copper (Cu) is similarly required for normal development and cellular bioenergetics [10]; in immunity, it functions as both a cofactor and a mediator of antimicrobial toxicity, and inadequate Cu availability compromises immune development and function [11]. Cu-dependent enzymes (including amine oxidases) also enable the utilization of nitrogen-containing substrates such as primary amines [12]. In this setting, Zn and Cu are dynamic signals at epithelial surfaces that shape antimicrobial defense, oxidative balance, and repair, and may influence vaginal barrier integrity [9, 13]. Through nutritional immunity, host tissues can restrict Zn to constrain microbial growth and mobilize Cu to damage invading bacteria and fungi, while pathogens counter with transport and detoxification systems [9, 13]. Despite this framework, cervicovaginal metal biology remains poorly characterized, and it is unclear whether local Zn and Cu vary across health and common vaginitis phenotypes or relate to symptoms and mucosal inflammation.

In a previous study, we evaluated the therapeutic potential of a zinc-containing hydroxyethyl cellulose (HEC)-based hydrogel for the management of genitourinary syndrome of menopause (GSM), demonstrating its suitability as a first-line treatment [14]. The zinc-containing vaginal hydrogel was an effective, topical, and nonhormonal treatment for GSM, providing significant symptom relief [14]. In addition, the zinc-containing vaginal hydrogel significantly improved vaginal health by alleviating different vulvovaginal symptoms. At the same time, its formulation provides a safe and patient-friendly alternative to oral therapies with no systemic side effects [15]. Hydrogel-based formulations are structured polymer networks designed to retain water and enable controlled release of active components. In the present study the zinc-containing vaginal hydrogel functioned as a localized hydroxyethyl cellulose-based delivery system composed of water, hydroxyethyl cellulose, zinc sulfate heptahydrate, and lactic acid. In vitro release and permeability data indicating that zinc is readily liberated from the gel matrix and remains bioavailable at the epithelial surface and enhance vaginal hydration and maintain normal vaginal zinc levels and an acidic pH conducive to vaginal health [14–16]. Hydrogels possess a three-dimensional crosslinked molecular structure that enables them to absorb substantial amounts of water and expand within a physiological environment [17]. This characteristic, which replicates the structure of natural tissues, makes hydrogels highly biocompatible and has attracted considerable attention in biomaterial applications over the past several decades [17–19]. We examined whether cervicovaginal lavage Zn and Cu concentrations differed between asymptomatic controls and women with symptomatic culture-defined phenotypes, including Candida albicans-positive and bacterial phenotypes. In an exploratory secondary analysis nested within the randomized trial, we also assessed whether these trace-element patterns varied according to allocation to fluconazole alone or fluconazole plus a zinc-containing vaginal hydrogel. We hypothesized that CVL Zn concentrations would differ across clinical and microbiological phenotypes, particularly in *C. albicans*-positive participants.

## Materials and methods

### Study design and participants

This report is a post hoc, exploratory secondary analysis of prospectively collected data from an ongoing, single-center, open-label randomized trial conducted at the outpatient clinic of the Department of Obstetrics and Gynecology, University of Debrecen, Hungary. Consecutive women assessed between June 2023 and April 2024 were screened. Eligibility required age ≥ 18 years and current vulvovaginal symptoms (e.g., discharge, malodor, pruritus, pain, dysuria, irritation, burning, dyspareunia). Exclusions were pregnancy, breastfeeding, abnormal cervical cytology within 12 months, recent antifungal and systemic antibiotic use, use of intra-uterine devices, including both hormone-releasing and copper-containing IUDs, known azole hypersensitivity, liver disease, and laboratory-confirmed sexually transmitted infections (Trichomonas vaginalis, Chlamydia trachomatis, Neisseria gonorrhoeae). In total, 56 participants were enrolled and scheduled for evaluations at baseline (week 0), week 4, week 8, and week 12. The study protocol was approved by the Hungarian National Institutional Review Medical Research Council (No. 3282-7/2023/EUIG), all participants gave written informed consent. The parent trial is registered at ClinicalTrials.gov (NCT05895162; 2023-05-15). This study adheres to CONSORT guidelines.

### Parent-trial procedures and clinical–microbiological classification

Women presenting with vulvovaginal symptoms underwent gynecological examination. At enrollment, symptoms were assessed using the Vulvovaginal Symptoms Questionnaire (VSQ-21) and the Candida Severity Score (CSS), and eligibility was determined based on these scores (VSQ-21 > 0 and CSS ≥ 3) together with clinical findings consistent with presumed vulvovaginal candidiasis. Before treatment initiation, cervicovaginal lavage (CVL) and vaginal culture samples were collected using a plastic speculum and plastic instruments to minimize exogenous metal contamination. Because final culture results were not available at enrollment, treatment and randomization were based on the clinical diagnosis. Participants were subsequently randomized 1:1 to receive either a single 150-mg oral dose of fluconazole alone or the same fluconazole regimen plus an intravaginal zinc-containing hydrogel (JUVIA; Fempharma LLC, Debrecen, Hungary). The hydrogel was self-administered at a dose of 2 mL daily for 2 weeks and then twice weekly for an additional 10 weeks. Its principal ingredients are water, hydroxyethyl cellulose, zinc sulfate heptahydrate at a concentration of 20 µM, and lactic acid. At each visit, demographic and clinical variables, including age, BMI, parity, recurrent infections, and interval treatments, were recorded. Vulvovaginal symptoms were assessed using the VSQ-21 and CSS [20, 21]. For the present exploratory analysis, participants were classified according to their visit-level clinical and microbiological findings. The *Candida albicans*-positive phenotype required vulvovaginal symptoms, a CSS ≥ 3, and a positive vaginal culture for *C. albicans*. The symptomatic bacterial-culture-positive phenotype required vulvovaginal symptoms and bacterial growth on routine vaginal culture, without implying a diagnosis of bacterial vaginosis or aerobic vaginitis. Asymptomatic controls had no vulvovaginal symptoms and a negative vaginal culture. Routine microbiological testing was performed according to institutional standards. The fungal-positive phenotype was restricted to symptomatic participants with routine vaginal culture confirming *Candida albicans;* non-albicans *Candida* species were not included in the analysis. The bacterial-positive phenotype was defined by vulvovaginal symptoms together with bacterial growth on routine vaginal culture. This category should not be interpreted as a formal diagnosis of bacterial vaginosis or aerobic vaginitis. The secondary analytic dataset did not contain Amsel criteria, Nugent scores, vaginal pH, whiff-test results, microscopy-based vaginosis scores, nucleic-acid amplification results, or species-level isolates. The bacterial culture-positive category was therefore not constructed from validated bacterial-vaginosis or aerobic-vaginitis criteria and should not be interpreted as either diagnosis. The dataset retained categorical fungal-growth and bacterial-growth results, species distributions and organism-specific mixed-infection analyses were consequently not performed.

### Cervicovaginal lavage (CVL) collection

After symptom assessment, a CVL sample was collected for Zn and Cu quantification by instilling 10 mL of sterile normal saline to bathe the vaginal walls and cervix for 60 s, with three successive aspirations and re-instillations using a plastic syringe, the pooled fluid was aspirated from the posterior fornix. Baseline CVL was obtained before any use of an intravaginal product. A standardized 48-hour washout period was required before each follow-up CVL collection, during which participants were instructed not to use the hydrogel. Samples were aliquoted into acid-washed polypropylene tubes, with field blanks (saline only) added intermittently, and stored at − 80 °C. For analysis, 3.00 mL was evaporated on an electric heating plate, the residue was wetdigested with 3 mL 65% HNO₃ and 1 mL 30% H₂O₂, then brought to 12 mL with ultrapure water (Synergy UV, Millipore), and filtered through 0.45 μm PVDF membranes. Zn and Cu were measured by ICP-OES (Agilent 5110 Vertical Dual View) using an SPS4 autosampler, a Meinhard^®^ nebulizer, and a double-pass spray chamber. Calibration used a seven-point, matrix-matched series prepared from 1000 mg/L spectroscopic stocks (ICP IV, Certipur, Merck) in 0.1 M HNO₃ with analytical-grade NaCl, accuracy was verified by independent standard recovery (± 5% acceptance), duplicates, and procedural blanks; limits of detection and quantification were defined as 3σ and 10σ of blanks, respectively. Instrument settings (view mode, RF power, nebulizer gas flow, analytical wavelengths) are provided in Tables 1 and 2.

**Table 1:** The operating parameters of ICP-OES

Parameter	Value
Pump speed (rpm)	15
Sample uptake time (sec)	15
Rinse time (sec)	30
Stabilization time (sec)	20
Nebulizer gas flow (L/min)	0.70
Number of repetitions	3

**Table 2:** The operating parameters and wavelength of ICP-OES

Element	Wavelength (nm)	Observational mode	Reading time (sec)
Zn	213.857	axial	10
Cu	324.754	axial	10

### Group definitions

The unit of analysis was the participant-visit, with repeated observations nested within participants. The culture-negative reference category comprised visit-level observations with negative fungal and bacterial cultures, it was not a separately recruited healthy or asymptomatic control cohort. Symptomatic culture-positive vaginitis required VSQ-21 > 0 and a positive vaginal culture. Fungal vaginitis required VSQ-21 > 0, CSS ≥ 3, a positive fungal culture, and a negative bacterial culture. Symptomatic culture-positive bacterial phenotypes required VSQ-21 > 0, a negative fungal culture, and a positive bacterial culture. Culture-defined fungal-positive and bacterial-positive categories used in separate exploratory comparisons were based on culture results irrespective of symptom scores. Phenotype categories were mutually exclusive within a visit, but the same participant could contribute observations to different categories over time.

### Statistical analysis

For each group and comparison, Zn and Cu concentrations were summarized using the mean and standard deviation (SD) on the original measurement scale for descriptive purposes. The number of unique participants contributing data (n) and the number of participant-visits were recorded. Distributions of Zn and Cu concentrations were evaluated using histograms, Q–Q plots, and skewness assessments. Both Zn and Cu demonstrated right-skewed distributions. Therefore, concentrations were log-transformed before inferential analyses to better approximate normality and stabilize variances. All model-based estimates were subsequently back-transformed to the original scale for presentation and interpretation. Arithmetic mean ± SD values on the original scale were retained as descriptive summaries only; hypothesis testing and confidence intervals were based on the log-transformed models and back-transformed GMRs. Primary analyses of Zn and Cu concentrations were conducted using linear mixed-effects models to account for repeated measurements within participants. Separate models were fitted for Zn and for Cu. Each model included: a random intercept for participant to account for within-subject correlation, fixed effects for group status (as defined above), time as a categorical variable (baseline, 4, 8, and 12 weeks), randomized hydrogel assignment (yes/no), and prespecified interaction terms where appropriate (e.g., group × time) to assess whether differences between groups varied across follow-up. Model parameters were estimated using restricted maximum likelihood. Model fit and assumptions were evaluated through residual diagnostics. In cases where mixed-effects models failed to converge or demonstrated singular random-effects structures, generalized estimating equations (GEE) with an exchangeable working correlation structure and robust (sandwich) standard errors were used as a sensitivity analysis. GEE results were interpreted as population-average effects. Adjusted differences between groups were estimated at the model level. For log-transformed outcomes, results were back-transformed and reported as geometric mean ratios (GMRs) with 95% confidence intervals (CIs). Effect direction was defined as higher or lower Zn or Cu concentration relative to the comparator group. Effect sizes were reported as GMRs derived from the model coefficients. Two-sided hypothesis tests were conducted for all prespecified contrasts. A p-value < 0.05 was considered statistically significant. A p-value ≥ 0.05 was interpreted as insufficient evidence of a difference and not as evidence of equivalence, absence of an association, or attenuation. Given the exploratory pilot nature of the study, no adjustment for multiple comparisons was applied, p-values should be interpreted as descriptive and hypothesis-generating. All analyses were conducted using validated statistical software IBM SPSS Statistics, version 29 (IBM Corp., Armonk, NY, USA).

## Results

**Fig. 1:**
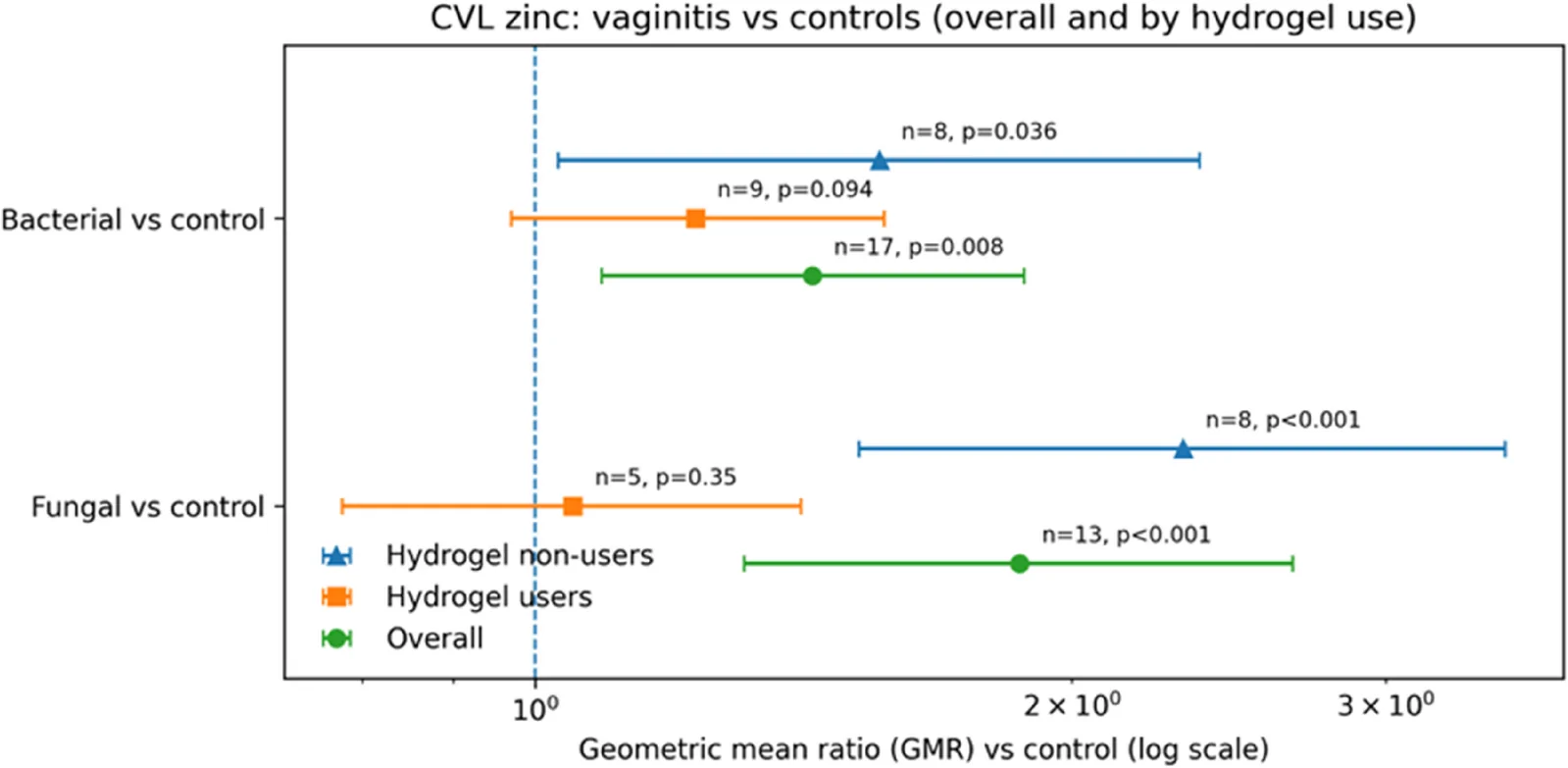
Forest plot of cervicovaginal lavage zinc (Zn) differences between vaginitis phenotypes and asymptomatic controls, shown overall and stratified by hydrogel exposure (GMR with 95% CI)

**Fig. 2:**
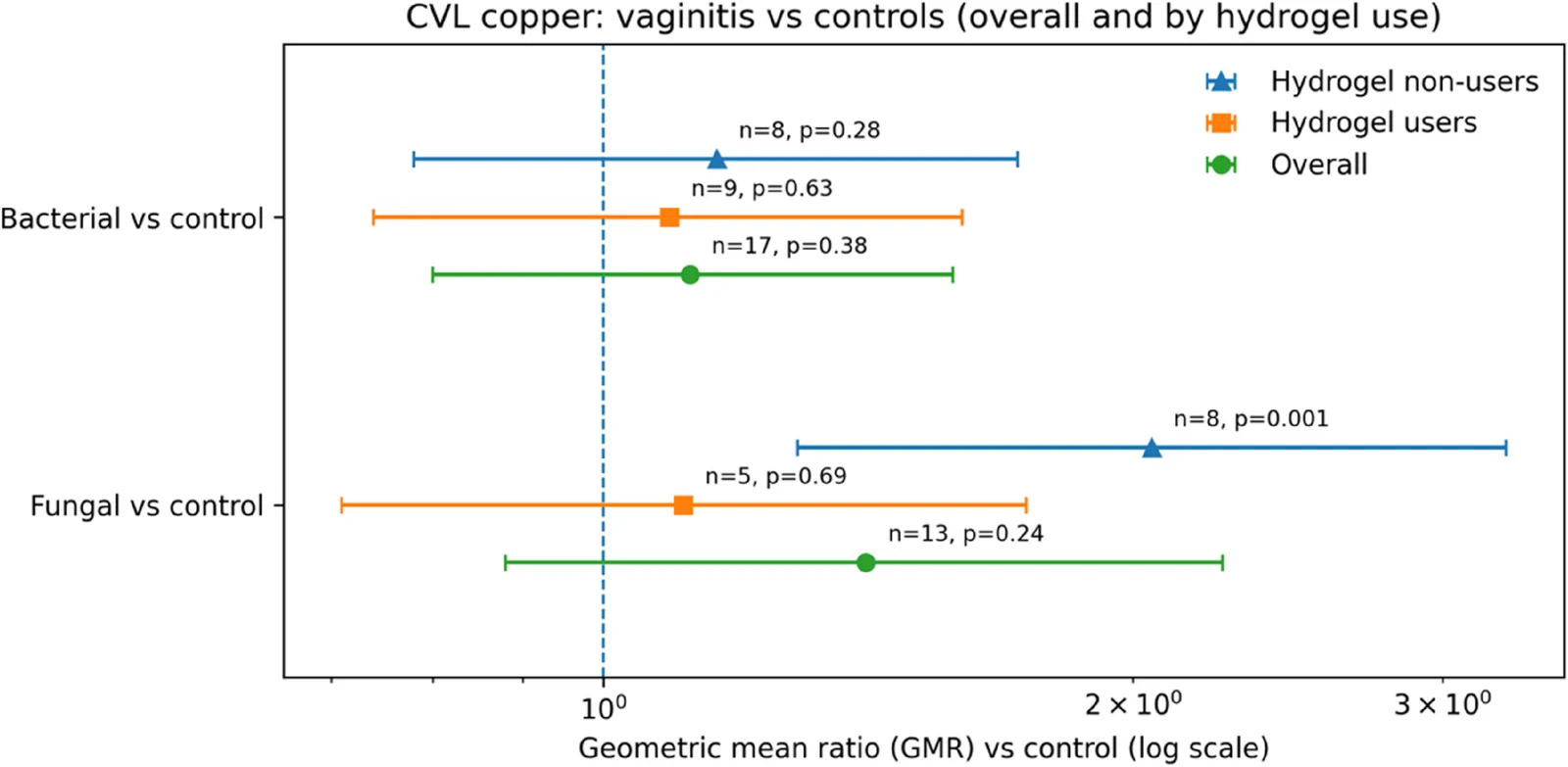
Forest plot of cervicovaginal lavage copper (Cu) differences between vaginitis phenotypes and asymptomatic controls, shown overall and stratified by hydrogel exposure (GMR with 95% CI)

Fifty-six women were enrolled in the study. The mean age was 36.0 ± 11.8 years, and the mean BMI was 23.1 ± 4.9 kg/m², indicating a predominantly young, normal-weight cohort. 19% had a history of vaginal delivery, 11% were postmenopausal, and 26% re-ported prior gynecological interventions (Table 3) In the overall (non-stratified) analysis, women with vaginitis had significantly higher CVL Zn concentrations compared with culture-negative women (Control) (mean ± SD: 99.4 ± 66.7 vs. 58.6 ± 27.2 µg/L, *p* < 0.001). In contrast, Cu concentrations did not differ significantly between groups (7.82 ± 4.36 vs. 6.03 ± 2.97 µg/L, *p* = 0.11). When stratified by infection subtype, women with fungal vaginitis demonstrated substantially higher Zn concentrations than Control (120.2 ± 95.6 vs. 58.6 ± 27.2 µg/L, *p* < 0.001), whereas women with symptomatic culture-positive bacterial phenotypes also had higher Zn levels (83.8 ± 55.2 vs. 58.6 ± 27.2 µg/L, *p* = 0.008). Cu concentrations were numerically higher in fungal vaginitis (10.2 ± 4.38 vs. 6.03 ± 2.97 µg/L) but did not reach statistical significance (*p* = 0.24); Cu concentrations in symptomatic culture-positive bacterial phenotypes were similar to Control (7.02 ± 4.99 vs. 6.03 ± 2.97 µg/L, *p* = 0.38) (Table 4). Culture-defined infection groups showed a similar pattern. Zn concentrations were higher in women with fungal-positive cultures compared with Control (84.6 ± 77.3 vs. 58.6 ± 27.2 µg/L, *p* = 0.071) and were significantly higher in women with bacterial-positive cultures (79.0 ± 53.3 vs. 58.6 ± 27.2 µg/L, *p* = 0.020). Cu concentrations did not differ significantly in either comparison. To evaluate whether the zinc-containing vaginal hydrogel modified trace-element profiles, analyses were repeated after stratification by randomized treatment assignment. Among zinc-containing hydrogel users, Zn concentrations in women with vaginitis were modestly higher than in Control (92.1 ± 58.4 vs. 67.4 ± 32.5 µg/L, *p* = 0.034). In contrast, among women who did not use the hydrogel, the difference in Zn concentrations was more pronounced (110.2 ± 77.1 vs. 54.2 ± 23.8 µg/L, *p* = 0.003). The estimated difference was numerically smaller; however, the study was not powered for a hydrogel-by-vaginitis interaction, and this pattern should be interpreted as hypothesis generating. Across both strata, Cu concentrations remained similar between vaginitis and Control. Among hydrogel users, no significant differences in Zn or Cu concentrations were observed between women with fungal vaginitis and Control (Zn: 69.1 ± 12.9 vs. 67.4 ± 32.5 µg/L, *p* = 0.35; Cu: 7.34 ± 1.24 vs. 6.63 ± 3.27 µg/L, *p* = 0.69). Given the fungal vaginitis subgroup size (*n* = 5) and the imprecision of the estimates, these non-significant findings should be interpreted as hypothesis-generating rather as evidence of no association or attenuation. Similarly, among hydrogel users with symptomatic culture-positive bacterial phenotypes, Zn and Cu concentrations did not differ significantly from Control (Zn: 83.2 ± 50.5 vs. 67.4 ± 32.5 µg/L, *p* = 0.094; Cu: 7.29 ± 5.74 vs. 6.63 ± 3.27 µg/L, *p* = 0.63) (Table 5). In contrast, among women who did not use the hydrogel, infection-associated differences in Zn concentrations were pronounced. Women with fungal vaginitis had markedly higher Zn concentrations compared with Control (154.3 ± 113.5 vs. 54.2 ± 23.8 µg/L, *p* < 0.001), accompanied by significantly higher Cu concentrations (12.13 ± 4.76 vs. 5.76 ± 2.86 µg/L, *p* = 0.001). Women with symptomatic culture-positive bacterial phenotypes who did not use the hydrogel also demonstrated higher Zn concentrations than Control (84.5 ± 61.7 vs. 54.2 ± 23.8 µg/L, *p* = 0.036), whereas Cu concentrations remained similar (6.66 ± 3.77 vs. 5.76 ± 2.86 µg/L, *p* = 0.28) (Table 6). Direct head-to-head comparisons showed no statistically significant difference in Zn concentrations between fungal vaginitis and symptomatic culture-positive bacterial phenotypes (120.2 ± 95.6 vs. 83.8 ± 55.2 µg/L, *p* = 0.66). Similarly, Zn concentrations did not differ between culture-defined fungal and bacterial groups (84.6 ± 77.3 vs. 79.0 ± 53.3 µg/L, *p* = 0.69). Cu concentrations were comparable across all phenotype-to-phenotype comparisons (Table 7). Figures 1 and 2 summarize the model-based differences in trace-element concentrations in cervicovaginal lavage between vaginitis phenotypes and controls. Specifically, the forest plots display geometric mean ratios (GMRs) with 95% confidence intervals for zinc (Fig. 1) and copper (Fig. 2), shown overall and stratified by zinc-hydrogel exposure. The dashed vertical line indicates no difference (GMR = 1), and labels provide the corresponding sample sizes and p-values for each comparison. Together, these figures visually confirm the direction and precision of the estimates reported in Tables 5, 6 and 7 and facilitate interpretation of subgroup variability across hydrogel users and non-users.

**Table 3:** Demographics and clinical characteristics: data are presented as numbers (%) or means ± standard deviations. BMI: mean body mass index

	(*N* = 56)
Age (years, mean ± SD)	36 ± 11.8
History of vaginal delivery (*n*,%)	11 (19)
Postmenopausal (*n*, %)	6 (11)
BMI (kg/m2, mean ± SD)	23.1 ± 4.9
History of gynecological intervention (*n*, %)	14 (26)

**Table 4:** Overall comparisons of CVL zinc and copper levels in women with vaginitis versus control. Arithmetic mean (SD) values are descriptive summaries on the original scale; GMRs, 95% CIs, and p-values are derived from the log-transformed longitudinal models. N denotes unique participants; analyses included all available participant-visits, with mutually exclusive phenotypic categories at each visit, although participants could change categories over time

Comparison	Marker	*N* (Group)	Mean (SD) Group	*N* (Control)	Mean (SD) Control	Effect (GMR)	95% CI	Direction	*p*-value	Model
Fungal vaginitis vs. Control	Zn	13	120.2 (95.6)	26	58.6 (27.2)	1.87	1.31–2.66	Higher	< 0.001	LMM
Culture-positive bacterial phenotype vs. Control	Zn	17	83.8 (55.2)	26	58.6 (27.2)	1.43	1.09–1.88	Higher	0.008	LMM
Fungal vaginitis vs. Control	Cu	13	10.2 (4.38)	26	6.03 (2.97)	1.41	0.88–2.25	Higher	0.24	LMM
Culture-positive bacterial phenotype vs. Control	Cu	17	7.02 (4.99)	26	6.03 (2.97)	1.12	0.80–1.58	Higher	0.38	LMM

**Table 5:** Zinc-containing vaginal hydrogel users: comparisons of CVL zinc and copper levels in women with vaginitis versus control. Arithmetic mean (SD) values are descriptive summaries on the original scale; GMRs, 95% CIs, and p-values are derived from the log-transformed longitudinal models. Non-significant p-values do not establish equivalence or attenuation. N denotes unique participants; analyses included all available participant-visits, with mutually exclusive phenotypic categories at each visit, although participants could change categories over time

Comparison	Marker	*N* (Group)	Mean (SD) Group	*N* (Control)	Mean (SD) Control	Effect (GMR)	95% CI	Direction	*p*-value	Model
Fungal vaginitis vs. Control	Zn	5	69.1 (12.9)	12	67.4 (32.5)	1.05	0.78–1.41	Higher	0.35	GEE
Culture-positive bacterial phenotype vs. Control	Zn	9	83.2 (50.5)	12	67.4 (32.5)	1.23	0.97–1.57	Higher	0.094	GEE
Fungal vaginitis vs. Control	Cu	5	7.34 (1.24)	12	6.63 (3.27)	1.11	0.71–1.74	Higher	0.69	GEE
Culture-positive bacterial phenotype vs. Control	Cu	9	7.29 (5.74)	12	6.63 (3.27)	1.09	0.74–1.60	Higher	0.63	GEE

**Table 6:** Zinc-containing vaginal hydrogel non-users: comparisons of CVL zinc and copper levels in women with vaginitis versus Control. Arithmetic mean (SD) values are descriptive summaries on the original scale; GMRs, 95% CIs, and p-values are derived from the log-transformed longitudinal models. Non-significant p-values do not establish equivalence or attenuation. N denotes unique participants; analyses included all available participant-visits, with mutually exclusive phenotypic categories at each visit, although participants could change categories over time

Comparison	Marker	*N* (Group)	Mean (SD) Group	*N*(Control)	Mean (SD) Control	Effect (GMR)	95% CI	Direction	*p*-value	Model
Fungal vaginitis vs. Control	Zn	8	154.3 (113.5)	14	54.2 (23.8)	2.31	1.52–3.50	Higher	< 0.001	LMM
Culture-positive bacterial phenotype vs. Control	Zn	8	84.5 (61.7)	14	54.2 (23.8)	1.56	1.03–2.36	Higher	0.036	LMM
Fungal vaginitis vs. Control	Cu	8	12.13 (4.76)	14	5.76 (2.86)	2.05	1.29–3.26	Higher	0.001	LMM
Culture-positive bacterial phenotype vs. Control	Cu	8	6.66 (3.77)	14	5.76 (2.86)	1.16	0.78–1.72	Higher	0.28	LMM

**Table 7:** Direct phenotype-to-phenotype comparisons. Arithmetic mean (SD) values are descriptive summaries on the original scale; GMRs, 95% CIs, and p-values are derived from the log-transformed longitudinal models. Non-significant p-values do not establish equivalence or attenuation. N denotes unique participants; analyses included all available participant-visits, with mutually exclusive phenotypic categories at each visit, although participants could change categories over time

Comparison	Marker	*N* (Group)	Mean (SD) Group	*N* (Control)	Mean (SD) Control	Effect (GMR)	95% CI	Direction	*p*-value	Model
Fungal vaginitis vs. Culture-positive bacterial phenotype	Zn	13	120.2 (95.6)	17	83.8 (55.2)	1.08	0.76–1.53	Higher	0.66	LMM
Fungal group vs. Bacterial group	Zn	18	84.6 (77.3)	20	79.0 (53.3)	1.05	0.80–1.38	Higher	0.69	LMM

## Discussion

In this prospective cohort of symptomatic women, cervicovaginal Zn and Cu concentrations showed an apparent, phenotype-associated pattern rather than a simple “high in disease, normal in health” relationship. Culture-positive vaginitis, particularly fungal vaginitis, was associated with higher Zn levels than controls. Fungal infections tended to show the highest values, with symptomatic culture-positive bacterial phenotypes showing intermediate levels. These findings suggest that, in acute inflammatory vaginitis, trace metal concentrations in CVL may increase, potentially reflecting exudation and local host responses, whereas in other gynecologic settings, reduced levels may reflect chronic epithelial compromise. This pattern complements our previous work in postmenopausal women, in which low CVL Zn was associated with vaginal atrophy and poor epithelial maturation, and CVL Zn increased after fractional CO₂ laser treatment, in parallel with symptom and colposcopic improvement [22]. In that context, low Zn marked hypoestrogenic, fragile mucosa. A tissue study from our group showed that vaginal Zn and Cu are tightly correlated and decline with age, supporting the concept of a Zn- and Cu-rich vaginal compartment modulated by estrogen status [23]. Together with the present data, this may support the concept of a spectrum. Low CVL Zn in atrophic, “under-nourished” epithelium versus high Zn and Cu in exudative, inflamed mucosa actively engaged in host–pathogen interactions. Our interventional studies with zinc-containing preparations fit this model. A zinc-containing vaginal moisturizer improved the VHI and symptoms in postmenopausal women [14]. In a separate trial, a zinc-containing hydrogel led to marked symptom improvement but nonlinear changes in CVL zinc, whereas oral Zn supple-mentation raised serum Zn without altering CVL Zn [15].

These findings indicate that cervicovaginal Zn is primarily locally regulated and does not simply mirror systemic status. The current study extends this by examining how the same zinc-containing hydrogel interacts with infection phenotype. Among women with vaginitis who did not receive the hydrogel, fungal infections had clearly higher Zn CVL levels. Among hydrogel users Zn concentrations in both fungal and symptomatic culture-positive bacterial phenotypes were similar to those observed in culture-negative women, while large Zn elevations were consistently observed only in the absence of hydrogel use. This attenuation was most pronounced in fungal vaginitis, where infection-associated increases in both Zn and Cu were evident among non-users but absent among hydrogel users. Collectively, these findings suggest that hydrogel use may be associated with an attenuation of infection-related Zn elevations, although this observation is exploratory and limited by small subgroup sizes. This suggests that vaginal use of zinc-containing hydrogel does not merely add more metal to an already enriched environment, but may support epithelial repair and reduce inflammatory exudation, thereby moderating the extreme metal signatures associated with candidiasis. However, the hydrogel-user fungal vaginitis subgroup included only five participants, the estimates were imprecise, and no formal hydrogel-by-phenotype interaction was demonstrated. Accordingly, this numerical pattern is hypothesis-generating and should not be interpreted as evidence that hydrogel use attenuated the infection-related trace-element response. Larger, appropriately powered studies are required to evaluate whether hydrogel assignment modifies local trace-element concentrations. Given the limited subgroup size, this remains exploratory, but it is compatible with a model in which symptom relief arises from subtle changes in metal handling and mucosal inflammation rather than from large shifts in total CVL Zn.

Experimental data on *Candida albicans* provide a biological framework. The zinc-binding protein Pra1 is induced under Zn restriction, drives neutrophil recruitment, and amplifies inflammation. Low-dose intravaginal Zn in mice down-regulates Pra1, reduces cytokine production, and prevents recurrent symptomatic candidiasis without lowering fungal burden [24]. The broader literature on Zn in RVVC is heterogeneous. Some studies report lower serum Zn in RVVC, suggesting mild systemic deficiency as a risk factor, whereas others find no consistent differences in serum or cervicovaginal Zn or correlation between the two [25, 26]. Our focus on acute, culture-confirmed infection and on local Zn and Cu patterns helps reconcile these results.

Systemic micronutrient status may shape long-term susceptibility, while local metal levels during an acute episode primarily reflect mucosal inflammation and exudation and can be modulated, but not fully normalized by intravaginal Zn therapy. Data from group B Streptococcus (GBS) models further support Zn as a bidirectional regulator. Zn deficiency increases the vaginal burden of GBS and alters the microbiome, whereas Zn can directly inhibit GBS growth, with strain-dependent resistance patterns [27–29]. These results echo our interpretation that low Zn compromises mucosal defenses, while locally increased Zn contributes to antimicrobial pressure and immune activation. Work on Cu intrauterine devices (Cu-IUDs) shows that chronic local Cu exposure is linked to increased bacterial diversity, loss of Lactobacillus, expansion of anaerobes, and higher rates of bacterial vaginosis compared with some hormonal methods. Cu-IUDs can promote more virulent, azole-resistant non-albicans Candida strains [30–34].

Our observation of higher CVL Cu in fungal vaginitis is consistent with Cu acting as a context-dependent modulator. Necessary for host defense but capable in excess or with chronic exposure of promoting dysbiosis and more aggressive fungal phenotypes. Overall, these data may point toward three provisional conclusions. First, Zn and Cu appear to contribute to vaginal “nutritional immunity,” potentially acting both as structural cofactors for epithelial and immune function and as levers to constrain or intoxicate microbes. Second, their roles seem to be context dependent. Low CVL Zn may be associated with atrophic, hypoestrogenic mucosa, whereas higher Zn and Cu may be linked to exudative, culture-positive vaginitis, particularly candidiasis. Third, systemic and local measurements are likely to provide different information; serum Zn may relate more to susceptibility and recurrence risk, while cervicovaginal Zn and Cu may better reflect the current mucosal state and its response to local therapies, such as zinc-containing hydrogels.

These interpretations should be considered preliminary, and larger, more detailed studies focused on underlying biological processes will be required to confirm and refine them. However, these findings can be viewed as a translational step linking compartment specific trace metal biology to an adjunct local intervention that could be feasibly integrated into routine care. Although current RVVC protocols typically rely on pro-longed oral fluconazole suppression, our data support the cautious hypothesis that ad-junctive local zinc delivery may contribute to clinical benefit when used alongside fluconazole, consistent with a potential host–pathogen–mucosa interaction. If confirmed in future randomized studies specifically powered for recurrence outcomes, this combined approach might also be associated with reduced recurrence. However, this possibility remains speculative and cannot be inferred from the present analysis.

The limitations of this study include its relatively small sample size, which may have reduced the ability to detect subtle differences. Given the sample size and subdivision into multiple vaginitis phenotypes, the study is primarily powered to detect moderate or larger differences in total Zn and Cu, and smaller effect sizes may not have been observable. Hydrogel-stratified phenotype subgroups were small, particularly fungal-positive vaginitis among hydrogel users (*n* = 5), which limited statistical power and precision, so any apparent attenuation of infection-related zinc elevations should be interpreted as exploratory and confirmed in larger cohorts. Therefore, non-significant subgroup findings should not be interpreted as evidence of no association. Mean ± SD values were reported on the original scale for descriptive consistency; because the distributions were right-skewed, these summaries may create an impression of greater precision than is warranted, and inferential conclusions were based on log-transformed models and back-transformed GMRs with 95% CIs. The small, heterogeneous cohort precluded reliable adjustment for age, menopausal status, and BMI; therefore, residual confounding, particularly by hormonal status, cannot be excluded. Additionally, the dilutional nature of cervicovaginal lavage sampling may introduce variability, as differences in dilution could potentially obscure true concentration changes. The observational nature of the analysis means that causality cannot be inferred.

The strengths of the study are a prospective design, standardized, metal-aware CVL collection, simultaneous Zn and Cu quantification with quality control, and integration of metal levels with symptoms, culture results, and infection phenotype. The nesting within a randomized trial of a zinc-containing hydrogel is an additional advantage allowing exploratory insight into how local Zn therapy may reshape metal patterns in bacterial versus fungal vaginitis.

Our findings contribute meaningfully to understanding trace-metal biology in the female genital tract. Cervicovaginal Zn and Cu emerge as integrative markers across a spectrum from postmenopausal atrophy to acute infectious vaginitis and as potential targets for local interventions. Future studies combining longitudinal metallomics, microbiome and mycobiome profiling, and more detailed assessment of metal speciation are needed to clarify causal links and to determine how best to harness Zn and Cu as biomarkers and therapeutic tools in the prevention and management of vaginitis. Randomized studies in women with recurrent vulvovaginal candidiasis are needed to test whether adjunct intravaginal zinc delivery alongside standard fluconazole regimens can meaningfully reduce recurrence risk and prolong time to relapse.

## Conclusions

Cervicovaginal Zn and Cu in our study behaved as flexible markers of vaginal health rather than fixed background values. Women with culture-positive vaginitis had higher Zn levels than controls, and fungal vaginitis showed the highest Zn and Cu concentrations. Together with our earlier findings in postmenopausal atrophy, this suggests a spectrum: low CVL Zn is linked to thin, hypoestrogenic mucosa with reduced trophicity, whereas higher Zn and Cu accompany inflamed, exudative mucosa during active infection. Collectively, these findings suggest that hydrogel use may be associated with an attenuation of infection-related Zn elevations, which should be interpreted as exploratory because subgroup sizes were small. Overall, our results indicate that Zn and Cu may serve as compartment-specific biomarkers of mucosal state and candidates for further evaluation as therapeutic targets in vaginitis. These findings also provide a cautious, hypothesis generating rationale for further evaluation of whether combining oral fluconazole with a zinc-containing intravaginal hydrogel could offer an additive clinical benefit, while any effect on recurrence remains to be demonstrated. 

## Supplementary Information

Supplementary Material 1.

## Data Availability

All data supporting the findings of this study are available within the paper and its Supplementary Information. We provide full access to the raw data upon request, which can be made by sending an email to the corresponding author’s email address.

## Abbreviations

BMI: Body Mass Index
Cu: Copper
CVL: Cervicovaginal Lavage
CSS: Candida Severity Score
GSM: Genitourinary syndrome of menopause
H₂O₂: Hydrogen Peroxide
HEC: Hydroxyethyl cellulose
HNO₃: Nitric Acid
ICP-OES: Inductively Coupled Plasma Optical Emission Spectrometry
NaCl: Sodium Chloride
SD: Standard Deviation
VSQ-21: Vulvovaginal Symptoms Questionnaire (21-item)
VVC: Vulvovaginal candidiasis
Zn: Zinc
