# Supplementary material for: Cervicovaginal zinc and copper profiles across vaginitis phenotypes and hydrogel assignment: an exploratory pilot study
Source: BMC Womens Health. 2026 Aug 1;26:566. doi: 10.1186/s12905-026-04768-6 (PMC13715171; doi:10.1186/s12905-026-04768-6)
Supplement: Supplementary file 1 [file 12905_2026_4768_MOESM1_ESM.docx]

**CONSORT 2010 checklist of information to include when reporting a randomised trial***

| **Section/Topic** | **Item No** | **Checklist item** | **Reported on page No** |
| --- | --- | --- | --- |
| **Title and abstract** | 1a | Identification as a randomised trial in the title | **N/A** |
|  | 1b | Structured summary of trial design, methods, results, and conclusions (for specific guidance see CONSORT for abstracts) | **2** |
| **Introduction** | | | |
| **Background and objectives** | 2a | Scientific background and explanation of rationale | **3-5** |
|  | 2b | Specific objectives or hypotheses | **5** |
| **Methods** | | | |
| **Trial design** | 3a | Description of trial design (such as parallel, factorial) including allocation ratio | **6-7** |
|  | 3b | Important changes to methods after trial commencement (such as eligibility criteria), with reasons | **N/A** |
| **Participants** | 4a | Eligibility criteria for participants | **6** |
|  | 4b | Settings and locations where the data were collected | **6** |
| **Interventions** | 5 | The interventions for each group with sufficient details to allow replication, including how and when they were actually administered | **7** |
| **Outcomes** | 6a | Completely defined pre-specified primary and secondary outcome measures, including how and when they were assessed | **5, 8-10** |
|  | 6b | Any changes to trial outcomes after the trial commenced, with reasons | **N/A** |
| **Sample size** | 7a | How sample size was determined | **6** |
|  | 7b | When applicable, explanation of any interim analyses and stopping guidelines | **N/A** |
| **Randomisation: sequence generation** | 8a | Method used to generate the random allocation sequence | 7 |
|  | 8b | Type of randomisation; details of any restriction (such as blocking and block size) | **7** |
| **Randomisation: allocation concealment mechanism** | 9 | Mechanism used to implement the random allocation sequence (such as sequentially numbered containers), describing any steps taken to conceal the sequence until interventions were assigned | **6-7** |
| **Randomisation: implementation** | 10 | Who generated the random allocation sequence, who enrolled participants, and who assigned participants to interventions | **6-7** |
| **Blinding** | 11a | If done, who was blinded after assignment to interventions (for example, participants, care providers, those assessing outcomes) and how | **6** |
|  | 11b | If relevant, description of the similarity of interventions | **7** |
| **Statistical methods** | 12a | Statistical methods used to compare groups for primary and secondary outcomes | **9-10** |
|  | 12b | Methods for additional analyses, such as subgroup analyses and adjusted analyses | **9-10** |
| **Results** | | | |
| **Participant flow (a diagram is strongly recommended)** | 13a | For each group, the numbers of participants who were randomly assigned, received intended treatment, and were analysed for the primary outcome | **6, 11** |
|  | 13b | For each group, losses and exclusions after randomisation, together with reasons | **N/A** |
| **Recruitment** | 14a | Dates defining the periods of recruitment and follow-up | **6** |
|  | 14b | Why the trial ended or was stopped | **6** |
| **Baseline data** | 15 | A table showing baseline demographic and clinical characteristics for each group | **20** |
| **Numbers analysed** | 16 | For each group, number of participants (denominator) included in each analysis and whether the analysis was by original assigned groups | **11-12, 21-25** |
| **Outcomes and estimation** | 17a | For each primary and secondary outcome, results for each group, and the estimated effect size and its precision (such as 95% confidence interval) | **11-13, 21-26** |
|  | 17b | For binary outcomes, presentation of both absolute and relative effect sizes is recommended | **N/A** |
| **Ancillary analyses** | 18 | Results of any other analyses performed, including subgroup analyses and adjusted analyses, distinguishing pre-specified from exploratory | **5-6, 11-13, 22-26** |
| **Harms** | 19 | All important harms or unintended effects in each group (for specific guidance see CONSORT for harms) | **11** |
| **Discussion** | | | |
| **Limitations** | 20 | Trial limitations, addressing sources of potential bias, imprecision, and, if relevant, multiplicity of analyses | **16-17** |
| **Generalisability** | 21 | Generalisability (external validity, applicability) of the trial findings | **16-18** |
| **Interpretation** | 22 | Interpretation consistent with results, balancing benefits and harms, and considering other relevant evidence | **13-18** |
| **Other information** | | | |
| **Registration** | 23 | Registration number and name of trial registry | **2, 6, 27** |
| **Protocol** | 24 | Where the full trial protocol can be accessed, if available | **N/A** |
| **Funding** | 25 | Sources of funding and other support (such as supply of drugs), role of funders | **27** |

*CONSORT 2010 checklist. The checklist should be interpreted together with the CONSORT 2010 Explanation and Elaboration and any relevant CONSORT extensions.
